# Supplementary material for: Urinary Protein Array Analysis to Identify Key Inflammatory Markers in Children with IgA Vasculitis Nephritis
Source: Children (Basel). 2022 Apr 27;9(5):622. doi: 10.3390/children9050622 (PMC9139281; doi:10.3390/children9050622)
Supplement: Supplementary file 1 [file children-09-00622-s001.zip › children-1640475-supplementary.pdf]

Urinary protein array analysis to identify key inflammatory markers in children with IgA Vasculitis nephritis

Julien Marro[1], Andrew J. Chetwynd[1], Rachael D. Wright[1], Silothabo Dliso[2], Louise Oni[1,3]

[1]Department of Women's and Children's Health, Institute of Life Course and Medical Sciences, University of Liverpool, Liverpool, United Kingdom;
[2]NIHR Alder Hey Clinical Research Facility, Clinical Research Division, Alder Hey Children's NHS Foundation Trust, Liverpool, United-Kingdom
[3]Department of Paediatric Nephrology, Alder Hey Children's NHS Foundation Trust Hospital, Liver-pool, United Kingdom

Supplementary materials – Tables S1-3

***Table S1:** Proteins assessed by R&Ds Systems Human Kidney Biomarker Array Kit ('Kit K') and Human XL Cytokine Array Kit ('Kit C').* 2
***Table S2**: Full results: Human Kidney Biomarker Array Kit* 6
***Table S3**: Full results: Human XL Cytokine Array Kit* 7

Table S1: Proteins assessed by R&Ds Systems Human Kidney Biomarker Array Kit ('Kit K') and Human XL Cytokine Array Kit ('Kit C').

**Name used**	**Full name**	**Alternative names**	**Assessed by**	
			**Kit K**	**Kit C**
Adiponectin	-	Arcp30	x	x
Aminopeptidase N	-	ANPEP	x	
Angiogenin	-	-		x
Angiopoietin-1	-	Ang1, ANGPT1		x
Angiopoietin-2	-	Ang-2, ANGPT2		x
AGT	Angiotensinogen	Serpin A8	x	
Annexin V	-	-	x	
ApoA1	Alipoprotein A	-		x
BAFF	B-cell activating factor	BLyS, TNFSF13B		x
BDNF	Brain-derived neurotrophic factor	Abrineurin		x
β2-Microglobulin	-	β2M	x	
C5/C5a	Complement component C5/C5a	-		x
CD14	Cluster of differentiation 14	-		x
CD30	Cluster of differentiation 30	TNFRSF8		x
CD31	Cluster of differentiation 31	PECAM-1		x
CD40 Ligand	Cluster of differentiation 40 ligand	CD154 TNFSF5		x
Chitinase-3-like-1	-	YKL-40		x
Clusterin	-	Apolipoprotein J	x	
CFD	Complement factor D	Adipsin		x
Cripto-1	-	TDGF-1		x
CRP	C-reactive protein	-		x
CXCL16	Chemokine (C-X-C motif) ligand 16	-	x	
Cyr61	Cysteine-rich angiogenic inducer 61	CCN1	x	
Cystatin C	-	CST3, ARMD11	x	x
Dkk-1	Dickkopf-related protein 1	SK, dickkopf WNT signalling pathway inhibitor 1		x
DPPIV	Dipeptidyl peptidase-4	CD26, DPP4, Dipeptidyl-peptidase IV	x	x
EGF	Epidermal growth factor	-	x	x
EGF-R	Epidermal growth factor receptor	ErbB1, HER1	x	
EMMPRIN	Extracellular matrix metalloproteinase inducer	Basigin, CD147		x
ENA-78	Epithelial-derived neutrophil-activating peptide 78	CXCL5		x
Endoglin		CD105, ENG		x

L-FABP	liver-type fatty acid-binding protein	FABP1	x	
Fas Ligand		CD95L, CD178, TNFSF6		x
Fetuin A		AHSG	x	
FGF-7	Fibroblast growth factor 7	KGF		x
FGF-19	Fibroblast growth factor 19	-		x
FGF-basic	Fibroblast growth-factor	FGF-2		x
Flt-3 Ligand	Fms-related tyrosine kinase 3 ligand	FLT3LG		x
G-CSF	Granulocyte colony-stimulating factor	CSF3		x
GDF-15	Growth/differentiation factor 15	MIC-1		x
GH	Growth hormone	Somatotropin		x
GM-CSF	Granulocyte macrophage colony-stimulating factor	CSF2		x
CXCL1	Chemokine (C-X-C motif) ligand 1 (CXCL1)	GROα, MSGA-α	x	x
HGF	Hepatocyte growth factor	Scatter factor, SF		x
ICAM-1	Intercellular Adhesion Molecule 1	CD54		x
IFN-γ	Interferon gamma	IFNG		x
IGFBP-2	Insulin-like growth factor-binding protein 2	-		x
IGFBP-3	Insulin-like growth factor-binding protein 3	-		x
IL-10	Interleukin-10	-	x	x
IL-11	Interleukin-11	-		x
IL-12p70	Interleukin-12p70	-		x
IL-13	Interleukin-13	-		x
IL-15	Interleukin-15	-		x
IL-16	Interleukin-16	-		x
IL-17A	Interleukin-17A	-		x
IL-18 BP	Interleukin-18 binding protein	IL-17, CTLA8		x
IL-19	Interleukin-19	-		x
IL-1α	Interleukin-1 alpha	IL-F1		x
IL-1β	Interleukin-1 beta	IL-F2		x
IL-1RA	Interleukin-1 receptor antagonist	IL-F3	x	x
IL-2	Interleukin-2	-		x
IL-22	Interleukin-22	IL-TIF		x
IL-23	Interleukin-23	IL-23A, SGRF		x
IL-24	Interleukin-24	C49A, FISP, MDA-7, MOB-5, ST16		x
IL-27	Interleukin-27	-		x
IL-3	Interleukin-3	-		x

IL-31	Interleukin-31	-		x
IL-32	Interleukin-32	-		x
IL-33	Interleukin-33	C9orf26, DVS27, NF-HEV		x
IL-34	Interleukin-34	C16orf77		x
IL-4	Interleukin-4	-		x
IL-5	Interleukin-5	-		x
IL-6	Interleukin-6	-	x	x
IL-8	Interleukin-8	CXCL8		x
IP-10	Interferon gamma-induced protein 10	CXCL10		x
I-TAC	Interferon-inducible T-cell alpha chemoattractant	CXCL11, SYCB9B		x
Leptin	-	OB		x
LIF	Leukemia inhibitory factor	-		x
NGAL	Neutrophil gelatinase-associated lipocalin	Lipocalin-2, LCN2, Siderocalin	x	x
MCP-1	Monocyte chemoattractant protein-1	CCL2, MCAF	x	x
MCP-3	Monocyte chemoattractant protein 3	CCL7, MARC		x
M-CSF	Macrophage colony-stimulating factor	CSF1		x
MIF	Macrophage migration inhibitory factor	-		x
MIG	Monokine induced by gamma interferon	CXCL9		x
MIP-1α/MIP-1β	Macrophage inflammatory proteins 1 alpha /1 beta	CCL3/CCL4		x
MIP-3α	Macrophage inflammatory protein 3 alpha	CCL20, Exodus-1, LARC		x
MIP-3β	Macrophage inflammatory protein 3 beta	CCL19, ELC		x
MMP-9	Matrix metallopeptidase 9	CLG4B, Gelatinase B	x	x
Myeloperoxidase	-	MPO, Lactoperoxidase		x
Neprilysin	-	CD10	x	
Osteopontin	-	OPN		x
PDGF-AA	Platelet-derived growth factor AA	-		x
PDGF-AA/BB	Platelet-derived growth factor AA/BB	-		x
Pentraxin 3	-	PTX3, TSG-14		x
PF4	Platelet factor 4	CXCL4		x
PSA	Prostate-specific antigen	KLK-3	x	x
RAGE	Receptor for advanced glycation endproducts	AGER	x	x

RANTES	Regulated on activation, normal T cell expressed and secreted	CCL5		x
RBP-4	Retinol Binding Protein 4	-	x	x
Relaxin-2	-	RLN2, RLXH2		x
Renin	-	Angiotensinogenase	x	
Resistin	-	ADSF, FIZZ3, RETN	x	x
SCF	Stem cell factor	-	x	
SDF-1α	Stromal cell-derived factor 1 alpha	CXCL12, PBSF		x
Serpin A3	-	α1-antichymotrypsin	x	
Serpin E1	-	PAI-I, PAI-1, Nexin		x
SHBG	Sex hormone binding globulin	ABP		x
ST2	Suppression of tumorigenicity 2	IL-1 R4, IL1RL1, ST2L		x
TARC	Thymus- and activation-regulation chemokine	CCL17		x
TfR	Transferrin	CD71, TFR1, TFRC, TRFR		x
TGF-α	Transforming growth factor alpha	TGFA		x
Thrombospondin-1	-	THBS1, TSP-1	x	x
KIM-1	Kidney injury molecule 1	TIM-1, HAVCR	x	
TIM-3	T-cell immunoglobulin and mucin-domain containing-3	HAVCR2		x
TNF R1	Tumour necrosis factor receptor 1	TNFRSF1A	x	
TNF-α	Tumour necrosis factor alpha	TNFSF1A	x	x
TFF3	Trefoil Factor 3	ITF, TFI	x	x
TWEAK	Tumor necrosis factor-like weak inducer of apoptosis	TNFSF12	x	
uPA	Urokinase-type plasminogen activator	Urokinase	x	
uPAR	Urokinase plasminogen activator receptor	PLAUR		x
VCAM-1	Vascular cell adhesion molecule 1	CD106	x	x
VEGF	Vascular endothelial growth factor	VPF		x
VEGF-A	Vascular endothelial growth factor-A	VPF-A	x	
Vitamin D BP	Vitamin D Binding Protein	VDB, DBP, VDBP		x

Table S2: Full results: Human Kidney Biomarker Array Kit

Protein	IgAVN vs IgAVwoN		IgAVN vs HC		IgAVwoN vs HC	
	Fold-change	p-value	Fold-change	p-value	Fold-change	p-value
Adiponectin	2.9919	0.57799	4.3938	0.27887	1.4686	0.32622
Angiotensinogen	6.4865	0.0096	22.603	0.007151	3.4846	0.054706
Annexin V	3.1693	0.45212	4.5046	0.37053	1.4213	0.90716
ANPEP	1.6726	0.93273	2.8759	0.47447	1.7194	0.2887
β2 microglobulin	1.1466	0.93184	3.1066	0.24131	2.7093	0.18944
Clusterin	1.7263	0.73026	3.8383	0.85582	2.2235	0.10659
CXCL16	3.728	0.013325	4.4656	0.013257	1.1979	0.58787
Cyr61	1.9483	0.5891	3.9752	0.41352	2.0403	0.83646
Cystacin-C	1.1894	0.97656	2.8185	0.29247	2.3698	0.17507
DPPIV	1.7591	0.61695	3.0626	0.19653	1.741	0.15967
EGF	1.0829	0.93131	3.0313	0.22998	2.7992	0.20843
EGF-R	5.8432	0.00232	7.8429	0.000424	1.3422	0.56969
FABP1	2.1775	0.44138	5.8558	0.13951	2.6892	0.42364
Fetuin-A	1.0103	0.62367	2.738	0.85618	2.71	0.1657
CXCL1	1.7859	0.53345	4.9731	0.20574	2.7846	0.53907
IL-10	1.5736	0.65926	4.8126	0.34642	3.0583	0.64184
IL-1RA	5.6892	0.12162	4.8312	0.095243	0.84918	0.51867
IL-6	2.5843	0.19587	5.5279	0.036444	2.139	0.49628
KIM-1	4.0891	0.003826	4.1496	0.00115	1.0148	0.93212
NGAL	1.1769	0.34533	1.8393	0.16901	1.5629	0.71228
MCP-1	3.3959	0.046906	11.956	0.001066	3.5206	0.001091
MMP-9	0.5616	0.93974	2.6748	0.42349	4.7628	0.45181
Neprilysin	1.7023	0.48842	2.3959	0.19983	1.4075	0.322
PSA	1.2936	0.7259	3.9295	0.45469	3.0377	0.71787
RAGE	0.82103	0.85485	1.689	0.50084	2.0571	0.40355
RBP4	1.0095	0.82887	2.2656	0.32605	2.2444	0.12205
Renin	1.2747	0.71454	3.7978	0.40608	2.9795	0.68952
Resistin	0.95591	0.9099	1.1464	0.40525	1.1993	0.44064
SCF	1.8794	0.3667	5.3492	0.19574	2.8463	0.98275
Serpin_A3	1.8348	0.9453	7.0708	0.40647	3.8537	0.2036
TFF3	1.1309	0.93257	2.8717	0.25503	2.5394	0.21273
Thrombospondin-1	1.383	0.71873	3.6518	0.39685	2.6405	0.67322
TNF-R1	0.9231	0.74193	1.7886	0.36378	1.9376	0.17036
TNF-α	1.6373	0.44583	2.6742	0.36577	1.6333	0.96245
TWEAK	2.4252	0.5586	4.1908	0.49631	1.728	0.96588
uPA	1.2215	0.88152	3.0317	0.20082	2.482	0.15934
VCAM-1	2.5729	0.22071	4.0982	0.15574	1.5928	0.3686
VEGF	1.2105	0.40523	1.5217	0.14103	1.257	0.34796

Table S3: Full results: Human XL Cytokine Array Kit

Protein	IgAVN vs IgAVwoN		IgAVN vs HC		IgAVwoN vs HC	
	Fold-change	p-value	Fold-change	p-value	Fold-change	p-value
Adiponectin	1.8287	0.57097	3.2492	0.17803	1.7768	0.21624
Angiogenin	1.6477	0.33875	2.212	0.11892	1.3425	0.13731
Angiopoietin-1	1.4268	0.32607	3.4687	0.033806	2.4312	0.35446
Angiopoietin-2	1.1577	0.7162	3.2967	0.05545	2.8476	0.12544
Apolipoprotein A 1	6.0461	0.020463	5.9723	0.034772	0.98779	0.58263
BAFF	9.7227	0.026053	26.468	0.003899	2.7223	0.078796
BDNF	1.0461	0.75692	2.6174	0.019991	2.502	0.063713
C5/C5a	4.5968	0.032956	6.5258	0.011829	1.4196	0.63215
CD14	2.0131	0.3988	2.9582	0.1626	1.4695	0.28197
CD30	0.52959	0.12288	0.75429	0.49646	1.4243	0.23274
CD31	0.86691	0.62807	1.7843	0.10109	2.0583	0.062136
CD40_Ligand	3.0402	0.044625	7.1976	0.007591	2.3675	0.070268
Chitinase 3-like 1	0.86917	0.75871	2.4447	0.10388	2.8127	0.37182
CFD	2.6001	0.008623	3.4104	0.004554	1.3116	0.40523
Cripto-1	7.8132	0.014841	7.1403	0.026301	0.91387	0.86666
CRP	2.4108	0.2663	3.0548	0.18704	1.2671	0.89643
Cystatin-C	1.2067	0.29366	1.1915	0.40639	0.98741	0.82491
Dkk-1	1.1093	0.56197	2.5345	0.011049	2.2847	0.1296
DPPIV	3.1747	0.1454	4.0274	0.075809	1.2686	0.4476
EGF	1.0446	0.9617	2.728	0.26843	2.6115	0.23998
EMMPRIN	1.0361	0.99501	2.001	0.25474	1.9313	0.21085
ENA-78	3.7452	0.058576	7.4035	0.015579	1.9768	0.11815
Endoglin	1.7528	0.020648	2.7075	0.014267	1.5447	0.19651
Fas Ligand	2.0645	0.086281	3.757	0.007516	1.8198	0.29808
FGF-19	1.5387	0.42579	3.2109	0.070494	2.0868	0.21824
FGF-7	1.4476	0.44304	2.9148	0.067729	2.0136	0.27496
FGF-basic	1.5811	0.29393	2.9534	0.068045	1.8679	0.50599
Flt-3 ligand	1.6921	0.13051	2.7008	0.018979	1.5961	0.22565
G-CSF	1.5053	0.21954	3.577	0.003326	2.3763	0.18161
GDF-15	1.5685	0.71341	2.3347	0.28713	1.4885	0.20007
GH	2.5913	0.067259	6.2705	0.003865	2.4198	0.017402
GM-CSF	1.4378	0.23444	3.2279	0.016377	2.245	0.3229
CXCL1	2.0059	0.022027	4.2743	0.000116	2.1309	0.016553
HGF	1.8013	0.02783	4.2975	0.00116	2.3858	0.019725
ICAM-1	4.0385	0.011899	10.249	6.39E-05	2.5377	0.14455
IFN-γ	1.2014	0.60944	3.2454	0.030262	2.7014	0.13751
IGFBP-2	1.7674	0.20262	4.8827	0.010683	2.7627	0.30689
IGFBP-3	3.4145	0.022753	10.73	2.13E-05	3.1426	0.11542
IL-10	1.6549	0.28815	3.3107	0.032085	2.0006	0.20312
IL-11	1.5168	0.44203	2.8766	0.098683	1.8965	0.30443

IL-12p70	1.0739	0.81759	3.0354	0.067595	2.8266	0.1222
IL-13	1.2101	0.60282	3.4298	0.016215	2.8343	0.049089
IL-15	1.4094	0.28861	3.153	0.01116	2.2371	0.18352
IL-16	1.2732	0.37424	2.7409	0.016	2.1528	0.27178
IL-17a	1.2603	0.87964	2.0202	0.31992	1.603	0.27878
IL-18 BP	1.0733	0.97381	2.6403	0.27038	2.4599	0.21658
IL-19	1.5401	0.31964	3.8165	0.008649	2.4781	0.029131
IL-1α	0.86362	0.87519	2.5854	0.10271	2.9936	0.084115
IL-1β	1.0638	0.72184	2.5271	0.0826	2.3755	0.21056
IL-1RA	1.2221	0.97565	2.3637	0.23858	1.9341	0.10512
IL-2	1.215	0.56018	2.6339	0.11002	2.1679	0.35638
IL-22	1.834	0.24804	4.4014	0.019852	2.3998	0.052279
IL-23	1.3567	0.4313	3.2976	0.02441	2.4307	0.16244
IL-24	1.2106	0.50425	3.1891	0.018619	2.6344	0.14649
IL-27	1.133	0.57041	3.2683	0.022913	2.8847	0.15347
IL-3	1.1007	0.57302	3.8068	0.074403	3.4585	0.32704
IL-31	1.3794	0.37148	3.2694	0.015051	2.3702	0.14672
IL-32	1.0852	0.55789	3.5242	0.004058	3.2475	0.079091
IL-33	1.3956	0.36308	3.7335	0.006638	2.6752	0.11432
IL-34	1.1551	0.55351	2.5888	0.025418	2.2413	0.20391
IL-4	1.989	0.077582	4.6257	0.003759	2.3257	0.035862
IL-5	2.029	0.046712	4.611	0.00292	2.2726	0.015636
IL-6	1.7949	0.29276	3.2726	0.051585	1.8233	0.19671
IL-8	1.9038	0.11659	2.8859	0.038825	1.5159	0.80768
IP-10	1.7876	0.12758	2.8273	0.022831	1.5816	0.12661
I-TAC	1.2475	0.37007	2.8928	0.008766	2.319	0.2042
PSA	1.0379	0.88677	2.5245	0.068415	2.4323	0.090241
Leptin	1.6779	0.2682	4.3158	0.00938	2.5722	0.022296
LIF	2.4099	0.00752	5.5375	0.001362	2.2978	0.06466
NGAL	1.0664	0.4986	2.063	0.10666	1.9345	0.77922
MCP-1	1.8851	0.06115	4.1117	0.00577	2.1812	0.13351
MCP-3	1.3311	0.37702	3.2094	0.016332	2.4111	0.17594
M-CSF	1.1701	0.28417	1.7828	0.08188	1.5236	0.1689
MIF	2.1612	0.015038	3.1333	0.006118	1.4498	0.29744
MIG	1.701	0.10966	3.8285	0.000975	2.2508	0.2024
MIP-1α/MIP-1β	1.4109	0.27706	2.5586	0.013899	1.8134	0.31822
MIP-3α	1.3186	0.34885	2.5306	0.017659	1.9192	0.2996
MIP-3β	0.92472	0.48424	3.488	0.012799	3.7719	0.32589
MMP-9	0.7438	0.72013	1.6703	0.087044	2.2456	0.49677
Myeloperoxidase	0.42741	0.3448	3.7338	0.059293	8.7358	0.88862
Osteopontin	1.2659	0.84319	2.1902	0.53528	1.7301	0.19047
PDGF-AA	0.95234	0.73517	2.2445	0.027002	2.3568	0.11891
PDGF-AA/BB	1.4861	0.23584	4.2025	0.007355	2.8278	0.13029
Pentraxin-3	1.1424	0.73195	3.0177	0.035324	2.6416	0.072243

PF4	2.8905	0.26081	7.528	0.035516	2.6044	0.16161
RAGE	0.61426	0.39621	1.2002	0.66954	1.954	0.27215
RANTES	1.9093	0.12788	4.4314	0.010905	2.321	0.1824
RBP-4	1.3193	0.70566	2.249	0.17302	1.7047	0.18646
Relaxin-2	1.2276	0.51042	2.909	0.048065	2.3697	0.26231
Resistin	1.5577	0.31074	1.4971	0.3415	0.96111	0.83688
SDF-1a	1.2941	0.2665	1.9324	0.070332	1.4933	0.23185
Serpin E1	2.491	0.033753	8.1136	9.91E-05	3.2572	0.069064
SHBG	7.5808	0.007742	13.339	0.00358	1.7595	0.24373
ST2	3.3299	0.014425	8.1713	0.000415	2.4539	0.082604
TARC	1.4684	0.33971	4.1432	0.010824	2.8215	0.073098
TFF3	1.0261	0.99463	2.7465	0.26291	2.6768	0.20413
TfR	1.9306	0.086845	5.6231	0.000308	2.9126	0.05055
TGF-α	0.8594	0.92868	2.2638	0.027643	2.6341	0.15107
Thrombospondin-1	0.91736	0.78108	2.6222	0.029302	2.8584	0.153
TIM-3	1.117	0.99997	2.6252	0.27146	2.3503	0.18122
TNF-α	1.1218	0.46899	2.3231	0.023407	2.0709	0.3851
uPAR	0.76021	0.44874	1.3151	0.33489	1.7299	0.15929
VCAM-1	2.3545	0.2753	4.2414	0.096912	1.8014	0.1735
VEGF	1.0948	0.79309	1.5399	0.22317	1.4066	0.28407
Vitamin D BP	2.5958	0.33743	4.3044	0.10077	1.6582	0.10032
